# ADS Guard: A Generalizable Defense Framework for Adversarially Robust Occupancy Detection in Smart Buildings

**DOI:** 10.3390/s26165039

**Published:** 2026-08-08

**Authors:** Pratiksha Chaudhari, Yang Xiao, Wei Sun

**Affiliations:** 1 Department of Computer Science, University of Alabama, Tuscaloosa, AL 35487-0290, USA; pgchaudhari@crimson.ua.edu; 2 School of Electrical Engineering and Automation, Hefei University of Technology, Hefei 230002, China; wsun@hfut.edu.cn

**Keywords:** occupancy detection, smart buildings, IoT sensors, devices, deep learning, machine learning, security, defense mechanism, adversarial attacks

## Abstract

Occupancy detection is fundamental to the operational intelligence of smart buildings, driving critical functions in energy management, HVAC automation, and physical security. While modern Deep Learning (DL) models have achieved high accuracy in parsing complex environmental sensor data, they remain highly vulnerable to adversarial examples, imperceptibly perturbed inputs designed to deceive neural networks. These vulnerabilities pose severe real-world risks, ranging from energy sabotage, in which systems heat empty rooms, to critical security breaches in which intruders go undetected. To address this security gap, we propose ADS-Guard, a novel Adversarial Detection and Sanitization (ADS) framework rooted in sequence-to-sequence autoencoder purification. Unlike standard denoising techniques, ADS-Guard incorporates a latent consistency regularization mechanism that encourages alignment between clean and adversarial representations in the latent feature space. We evaluated ADS-Guard using a comprehensive experimental pipeline comprising five distinct DL architectures (LSTM, GRU, 1D-CNN, MLP, and Transformer) across three diverse datasets: (1) The UCI Occupancy dataset (20,699 samples) for standard binary detection; (2) Building59 dataset (7200 samples) for three-class occupancy-level classification (Low, Medium, High); and (3) Room Occupancy dataset (10,129 samples), representing a highly imbalanced binary occupancy-detection task. We evaluate ADS-Guard against both Fast Gradient Sign Method (FGSM) and Projected Gradient Descent (PGD) attacks across diverse occupancy datasets and model architectures. We further assess the framework under adaptive white-box attacks and compare its performance with FGSM-based and PGD-based adversarial training baselines. Our results demonstrate that adversarial attacks can substantially degrade occupancy-detection performance across datasets and model architectures. ADS-Guard consistently improves robustness relative to undefended models against both FGSM and PGD attacks, recovering a substantial portion of the lost performance in binary occupancy tasks and providing meaningful gains in the more challenging multi-class setting. Furthermore, ADS-Guard remains effective under stronger adaptive threat models while providing a practical retraining-free defense that can be integrated with existing occupancy-detection systems without modifying downstream classifiers.

## 1. Introduction

The transition of the built environment from static, reactive physical structures into intelligent, cognitive ecosystems represents one of the most significant engineering evolutions of the last decade. At the core of this transformation is occupancy detection, the ability of a building to autonomously perceive, interpret, and anticipate human presence in real time. This capability has moved beyond simple motion sensing to become the operational backbone of modern facility management, driving critical decision-making across energy efficiency, HVAC automation, and physical security [1,2]. With the building sector currently accounting for approximately one-third of global energy consumption and a significant share of energy-related carbon emissions [3], the precision of these detection systems is directly linked to global sustainability goals [4]. By shifting from fixed, static operational schedules to dynamic, demand-driven control, smart buildings can achieve massive efficiency gains, reducing energy waste by up to 30% without compromising occupant comfort [5].

To achieve this level of granularity, modern smart buildings rely on a complex sensor fusion approach that integrates heterogeneous data streams from temperature, humidity, CO_2_, light, and acoustic sensors [6,7]. The interpretation of this high-dimensional, multivariate time series data has increasingly been delegated to Deep Learning models [8]. Advanced architectures, ranging from Recurrent Neural Networks to Transformers, have demonstrated exceptional proficiency in capturing the non-linear temporal dependencies inherent in human occupancy patterns [9,10]. Consequently, the research community has made significant strides in addressing the first two pillars of the Smart Building Trilemma: achieving high Accuracy through complex model architectures, and ensuring Privacy by utilizing non-intrusive environmental sensors rather than cameras [6]. However, as these AI systems are granted autonomous control over critical physical infrastructure, they expose a third, often dangerously neglected dimension of the trilemma: Security [11,12].

The integration of deep learning into critical control loops has introduced a vulnerability that is fundamentally different from traditional cybersecurity threats. While conventional IT security focuses on preventing unauthorized network access or data exfiltration, the threat to AI-driven occupancy detection targets the model’s perception of reality itself [10]. Recent literature in computer vision has extensively documented that deep neural networks are inherently brittle; adversarial examples can easily deceive them, inputs containing carefully crafted, imperceptible perturbations designed to maximize prediction error [13]. In the context of a smart building, this vulnerability transforms a software flaw into a physical and financial threat. We identify two primary attack vectors that exploit this fragility. The first is Energy Sabotage, in which an attacker introduces subtle electromagnetic or digital noise into sensor readings, tricking the system into detecting occupancy in an empty zone and forcing high-consumption HVAC and lighting systems to operate at full capacity unnecessarily [14]. Second, and perhaps more critical, is the Security Breach scenario. Here, a sophisticated intruder could manipulate environmental signals to mimic the statistical signature of an empty room, effectively rendering AI-driven surveillance and alarm systems blind to unauthorized access [11].

Despite the severity of these potential outcomes, the intersection of adversarial defense and smart building occupancy detection remains virtually unexplored in the current literature [15,16]. This represents a significant research gap. While adversarial robustness has been studied extensively in the domains of Computer Vision and Natural Language Processing, the defense mechanisms developed for static images or discrete text are ill-suited for the continuous, multivariate nature of environmental sensor data. Time series data is characterized by unique challenges such as sensor drift, varying sampling rates, and complex temporal correlations that standard spatial defenses cannot address. Furthermore, the existing IoT security research has predominantly focused on hardening specific model architectures. This approach is impractical in the heterogeneous landscape of edge computing where diverse models, from lightweight MLPs to complex Transformers, coexist. Despite growing interest in adversarial robustness for time-series learning, comparatively little attention has been given to input-level defenses for occupancy detection systems operating on multivariate environmental sensor streams. The existing approaches primarily focus on model-specific hardening strategies, while comparatively few studies investigate retraining-free purification mechanisms applicable across heterogeneous occupancy detection architectures.

To address this critical void, this paper shifts the defense paradigm from model-specific hardening to universal input purification. We propose ADS Guard, Adversarial Detection and Sanitization Guard, a novel defense framework specifically tailored for the temporal dynamics of smart building data. The core hypothesis of ADS Guard is that while adversarial perturbations are highly effective in shifting data points in the raw signal space, they fail to preserve semantic consistency in the latent feature space. To leverage this, ADS Guard utilizes a sequence-to-sequence autoencoder trained with a unique Latent Consistency Regularization objective [17]. Unlike traditional autoencoders that simply minimize reconstruction error, our model is explicitly trained to ensure that both clean and adversarially perturbed inputs map to the same latent representation. This encourages the purifier to suppress adversarial perturbations while preserving the semantic and temporal structure of the original signal, thereby improving the robustness of downstream classifiers without requiring modifications to their architectures or training procedures [9,12].

We validate this framework through one of the most comprehensive experimental studies in this domain. To prove the universality of our defense, we evaluate ADS Guard across three distinct data domains representing the full spectrum of deployment challenges: the standard binary UCI Occupancy dataset [18], a multi-class Building59 dataset distinguishing varying usage levels [19], and a highly imbalanced Room Occupancy dataset representing rare event detection [20]. Furthermore, we test the defense against five distinct deep learning architectures, LSTM, GRU, 1D CNN, MLP, and Transformer, subjected to both single-step (FGSM) and iterative (PGD) attacks. This multi-model, multi-dataset evaluation, together with adaptive attack analysis and comparisons against adversarial-training baselines, demonstrates that an input-purification framework can substantially improve robustness against adversarial perturbations across diverse occupancy-detection settings.

To the best of our knowledge, this work is among the earliest studies to systematically evaluate adversarial attacks and defense mechanisms across multiple occupancy-detection datasets, architectures, and deployment scenarios. In addition to standard FGSM and PGD evaluations, we investigate adaptive white-box attacks that explicitly optimize through the ADS-Guard purification module. We further benchmark ADS-Guard against FGSM-based and PGD-based adversarial training baselines and examine the contribution of latent consistency regularization through a comprehensive ablation study.

Our key contributions are as follows:

- We introduce a classifier-independent defense mechanism rooted in sequence-to-sequence autoencoder purification. Unlike standard denoising methods that operate solely in the raw signal space, ADS-Guard incorporates a unique Latent Consistency Regularization objective. This mechanism enforces semantic alignment between clean and perturbed inputs within the latent feature manifold, enabling the effective sanitization of sensor data while preserving the temporal context required for accurate occupancy detection.
- We define the adversarial threat landscape for the built environment, categorizing attack impacts into two distinct physical outcomes: “Energy Sabotage” (induced false positives that lead to resource wastage) and “Security Breaches” (induced false negatives that lead to undetected intrusion). We utilize this framework to quantify the vulnerability of occupancy systems against both single-step (FGSM) and iterative (PGD) gradient-based attacks, providing a systematic evaluation of occupancy-detection vulnerabilities under gradient-based adversarial attacks.
- We evaluate ADS-Guard as an input-level defense mechanism that operates independently of the downstream classifier. Through extensive testing on five distinct deep learning architectures, LSTM, GRU, 1D-CNN, MLP, and Transformer, we demonstrate that the defense can improve robustness across diverse classifier architectures without requiring retraining, fine-tuning, or modification of the underlying predictive models, a critical feature for scalable IoT deployment.
- We conduct a comprehensive evaluation across three datasets representing fundamentally different occupancy-detection tasks: standard binary detection (UCI Occupancy), multi-class usage classification (Building59), and highly imbalanced rare-event monitoring (Room Occupancy). This validates ADS-Guard’s robustness across diverse data distributions and class structures, ensuring its reliability in heterogeneous real-world environments.

The remainder of this paper is organized as follows: Section 2 provides a review of related work, synthesizing the current literature on occupancy detection techniques and the emerging field of adversarial security in time-series data. Section 3 presents the problem formulation and adversarial threat model. Section 4 describes the ADS-Guard framework and latent consistency regularization mechanism. Section 5 explains the experimental setup, detailing the three datasets and five model architectures; and Section 6 presents a comprehensive analysis of the results. Finally, Section 7 concludes the paper, summarizing the key findings and outlining potential directions for future research in secure smart building technologies.

## 2. Related Work

This section critically reviews the state of the art across three converging domains: data-driven occupancy detection in smart buildings, adversarial vulnerabilities in time-series data, and defense mechanisms for deep learning models. We highlight the specific limitations of current methodologies to delineate the research gap that the ADS-Guard framework addresses.

*Evolution of Deep Learning in Smart Buildings* The paradigm of occupancy detection has transitioned significantly from hardware-centric reliance on Passive Infrared (PIR) sensors, which suffer from high false-negative rates when occupants are stationary, to data-centric environmental sensing. Candanedo and Feldheim [21] established the foundational benchmark in this domain (the UCI Occupancy dataset), demonstrating that non-intrusive variables such as temperature, humidity, light, and CO_2_ could be fused to accurately infer human presence using statistical models like Support Vector Machines (SVM) and Random Forests. While effective, these early methods relied heavily on manual feature engineering, limiting their scalability in dynamic environments.

However, the non-linear and time-dependent nature of thermal dynamics necessitated a shift toward Deep Learning (DL). First-generation DL approaches focused on Convolutional Neural Networks (CNNs). Wang et al. [22] demonstrated that 1D-CNNs and Fully Convolutional Networks (FCNs) could outperform traditional baselines by acting as effective feature extractors for sliding windows of sensor data. Second-generation approaches integrated Recurrent Neural Networks (RNNs), specifically LSTMs and GRUs, to manage the “vanishing gradient” problem and model long-term historical context [23,24]. Most recently, the field has adopted Transformer-based architectures. Zerveas et al. [25] introduced a framework for multivariate time-series representation learning using self-attention, proving that Transformers can capture global dependencies that RNNs miss.

Despite this trajectory toward increasingly complex architectures to maximize *Accuracy*, the literature has largely treated these models as “black boxes” functioning in benign environments. The robustness of these high-performing architectures against malicious adversarial interference remains largely unexamined in the specific context of building automation.

*Adversarial Threats in the Time-Series Domain.* The concept of “adversarial examples,” inputs intentionally perturbed to maximize model error, was formalized by Szegedy et al. [13] and Goodfellow et al. [26] in the context of computer vision. While the threat is well-understood in image processing, its application to time-series data presents unique challenges regarding imperceptibility and temporal coherence.

Fawaz et al. [27] conducted the first comprehensive survey of adversarial attacks on Deep Time Series Classifiers (DTSC), revealing that architectures considered robust in other domains are brittle when applied to 1D signals. In the IoT domain, researchers have begun to explore these threats; for example, studies on smart home event detection have shown that “semantic” attacks can mislead sensor systems [28]. We focus on two primary gradient-based attack vectors in this work:

- FGSM (Fast Gradient Sign Method): A single-step attack proposed by Goodfellow et al. [26] that linearizes the loss function to generate rapid perturbations. It serves primarily as a baseline for vulnerability scanning.
- PGD (Projected Gradient Descent): An iterative variation proposed by Madry et al. [29] that applies perturbations in small steps, projecting the result back onto the ϵ-ball. It is currently regarded as the “strongest first-order adversary” and serves as a worst-case scenario for security evaluations.

*Defenses and the “Purification” Gap.* Current defense mechanisms generally fall into two categories: *Adversarial Training* and *Input Pre-processing*. Adversarial training, as championed by Madry et al. [29], involves solving a minimax optimization problem in which the model is trained on adversarial examples generated on the fly. Recent studies continue to regard adversarial training as one of the strongest empirical defenses against gradient-based attacks. By exposing the classifier to adversarial examples during training, adversarial training improves robustness under both FGSM and PGD attacks. However, this robustness comes at the cost of increased training complexity, attack-specific optimization, and the requirement that the original training data and model parameters remain available for retraining. These requirements may limit its practicality in deployed smart-building systems where occupancy models have already been integrated into operational control pipelines. While effective, this approach suffers from the "robustness-accuracy trade-off," often degrading performance on clean data [30]. Furthermore, retraining large models on edge devices is computationally prohibitive. Consequently, adversarial training serves as an important benchmark for evaluating whether retraining-free purification methods can achieve meaningful improvements in robustness while avoiding the deployment challenges associated with classifier retraining.

A more modular alternative is *Input Purification*, typically using Denoising Autoencoders (DAEs) to sanitize data before classification. Meng and Chen [31] proposed “MagNet,” which detects adversarial examples by measuring their distance from the data manifold. However, standard DAEs minimize simple reconstruction error (MSE). As noted by [32] and recent critiques [33], this can lead to a “masking effect” in which the autoencoder unwittingly reconstructs the adversarial noise if it is sufficiently subtle, thereby failing to remove the threat.

This identifies the specific gap our work addresses: a lack of defense mechanisms that enforce semantic consistency rather than merely perform pixel-level reconstruction. ADS-Guard bridges this gap by introducing a Latent Consistency loss that forces the encoder to map adversarial inputs to the same feature representation as clean inputs, encouraging attack-invariant latent representations that improve robustness during reconstruction.

While adversarial training and purification-based defenses have been extensively studied in image classification, comparatively few works have examined these strategies in occupancy-detection systems operating on multivariate environmental sensor streams. Furthermore, the existing studies rarely evaluate defenses across multiple occupancy datasets, model architectures, and attack settings. This motivates a systematic investigation of adversarial robustness and purification-based defenses in smart-building environments.

To systematically position our contributions within the broader IoT security landscape, we provide a structured comparison in Table 1 and Table 2. We contrast Occupancy Detection against three analogous time-series domains where adversarial threats have been more aggressively explored: Smart Grids, Medical Diagnostics (ECG), and Human Activity Recognition (HAR).

As illustrated, while domains such as Smart Grids and ECG have established “Safety-Critical” threat models, Building Occupancy remains comparatively underexplored from an adversarial robustness perspective, despite its influence on building-control decisions. ADS-Guard is designed to address this gap by providing a broadly applicable purification framework to enhance adversarial robustness in occupancy-detection systems that rely on building environmental sensor data.

## 3. Problem Formulation and Threat Model

We formulate building occupancy inference from environmental sensors as a supervised multivariate time series classification problem. Let

(1) $$\begin{array}{c} X=x_{1},x_{2},\ldots ,x_{T},\;x_{t}\in R^{F} \end{array}$$

denote a sequence of sensor observations over a temporal window of length *T*, where *F* is the number of sensor features, such as temperature, humidity, CO_2_, light intensity, and energy-related signals. Given this input sequence, the objective is to learn a function

(2) $$\begin{array}{c} f_{\theta }:R^{T\times F}\to Y, \end{array}$$

parameterized by θ, that maps the sequence X to an occupancy label y∈Y. Depending on the deployment scenario, Y may represent a binary label space (e.g., occupied vs. empty) or a multi-class space (e.g., low, medium, high occupancy).

In practical smart building systems, $f_{\theta }\left(\cdot \right)$ is instantiated with deep learning architectures that model temporal dependencies and cross-sensor correlations, including recurrent neural networks, convolutional temporal models, and attention-based Transformers [22,23,24,25]. Under normal operating conditions, the predicted label

(3) $$\begin{array}{c} \hat{y}=f_{\theta }\left(X\right) \end{array}$$

achieves high accuracy and enables downstream control logic to drive energy management, HVAC automation, and security monitoring.

### 3.1. Adversarial Threat Model

We consider a white-box adversarial setting in which an attacker has full knowledge of the target model architecture, parameters, and deployed defense mechanisms, including any deployed purification mechanism, such as ADS-Guard [26,29]. The adversary can inject carefully crafted perturbations into the sensor input stream and optimize those perturbations using gradient information obtained from either the classifier alone or the combined purifier-classifier pipeline. The adversary’s goal is to construct an adversarial sequence $X^{adv}$ such that

(4) $$\begin{array}{c} X^{adv}=X+\delta , \end{array}$$

where δ is a bounded perturbation satisfying

(5) $$\begin{array}{c} {∥\delta ∥}_{\infty }\leq \epsilon , \end{array}$$

and yet causes the classifier to output an incorrect prediction, i.e.,

(6) $$\begin{array}{c} f_{\theta }\left(X^{adv}\right)\neq y. \end{array}$$

All perturbation budgets reported in this work are applied after feature standardization. Consequently, the parameter ϵ denotes the maximum perturbation measured in standardized feature units. We focus on *untargeted* attacks, where the objective of the adversary is to maximize the classification loss rather than force a specific target class [26,29,37]. Let $L(f_{\theta }\left(X\right),y)$ denote the training loss function (binary cross-entropy for binary occupancy datasets and sparse categorical cross-entropy for the multi-class Building59 dataset). The adversary seeks a perturbation δ that maximizes this loss under the $\ell_{\infty }$ constraint.

### 3.2. Fast Gradient Sign Method (FGSM)

The Fast Gradient Sign Method (FGSM) is a single-step, first-order adversarial attack that constructs adversarial examples by moving the input in the direction of the loss gradient [26]. Formally, the adversarial sequence is generated as

(7) $$\begin{array}{c} X^{adv}=X+\epsilon \cdot \mathrm{sign}\left(\nabla_{X}L\left(f_{\theta }\left(X\right),y\right)\right), \end{array}$$

where ϵ>0 controls the perturbation magnitude. Despite its simplicity, FGSM is effective at exposing the vulnerability of deep models to small, carefully aligned perturbations and serves as a strong baseline for robustness evaluation [13].

### 3.3. Projected Gradient Descent (PGD)

Projected Gradient Descent (PGD) is an iterative, multi-step extension of FGSM that is widely regarded as a stronger, first-order adversary [29,38]. Starting from an initial point $X_{0}^{adv}=X$, the attack iteratively updates the adversarial example according to

(8) $$\begin{array}{c} X_{k+1}^{adv}=\Pi_{B_{\epsilon }\left(X\right)}\left(X_{k}^{adv}+\alpha \cdot \mathrm{sign}\left(\nabla_{X}L\left(f_{\theta }\left(X_{k}^{adv}\right),y\right)\right)\right), \end{array}$$

where α is the step size, and ΠBϵ(X)(·) denotes projection onto the ℓ∞-ball of radius ϵ centered at the original input X. This projection ensures that the final perturbation remains within the allowed budget. By iteratively refining the perturbation, PGD typically produces substantially stronger adversarial examples than single-step methods.

### 3.4. Attack Surface in Smart Building Systems

In smart building environments, adversarial perturbations can be realized through multiple physical and cyber-physical channels, including sensor spoofing, electromagnetic interference, or subtle digital manipulation of transmitted measurements [11,12,14,39,40]. Unlike attacks targeting network infrastructure or stored data, these attacks directly compromise the model’s perception of the physical environment. As a result, small, carefully crafted changes to temperature, ${\mathrm{CO}}_{2}$, humidity, or energy readings can propagate through the learning system and trigger incorrect operational decisions.

While the adversarial perturbations considered in this study are generated digitally, their physical realization may vary, depending on the deployment environment. Coordinated perturbations across multiple heterogeneous sensors can be challenging to achieve in practice. They may require sensor spoofing, compromised building-management systems, wireless communication attacks, or direct manipulation of environmental conditions. Consequently, the attack scenarios investigated in this work should be interpreted as worst-case security evaluations intended to characterize model vulnerability under bounded perturbations rather than direct simulations of a specific physical attack.

Given the heterogeneous nature of smart building deployments, where multiple model architectures may coexist on edge and cloud platforms, we do not assume the ability to modify or retrain the downstream classifier for each defense scenario. Instead, we treat the classifier $f_{\theta }$ as a fixed decision module and focus on defending the input space.

Formally, our objective is to design a purification function

(9) $$\begin{array}{c} P:R^{T\times F}\to R^{T\times F} \end{array}$$

such that, for a potentially adversarial input $X^{adv}$, the purified sequence

(10) $$\begin{array}{c} \tilde{X}=P\left(X^{adv}\right) \end{array}$$

satisfies

(11) $$\begin{array}{c} f_{\theta }\left(\tilde{X}\right)=f_{\theta }\left(X\right), \end{array}$$

while preserving the semantic and temporal structure of the original sensor signal. This formulation motivates an input-level defense based on sequence purification, which aims to remove adversarial artifacts before the data reaches the downstream decision logic.

### 3.5. Adaptive White-Box Attacks

Traditional FGSM and PGD attacks assume that adversarial examples are generated directly against the target classifier. However, when a defense mechanism is deployed, a knowledgeable adversary may attempt to optimize perturbations through the defense itself. Such attacks are commonly referred to as adaptive white-box attacks and are considered a more rigorous evaluation of defense robustness [29,33].

Let P(·) denote the ADS-Guard purification function and let $f_{\theta }\left(\cdot \right)$ denote the downstream occupancy classifier. Under an adaptive threat model, the attacker optimizes perturbations against the composite system.

(12) $$\begin{array}{c} g\left(X\right)=f_{\theta }\left(P\left(X\right)\right). \end{array}$$

The adversarial objective becomes

(13) $$\begin{array}{c} \max_{{∥\delta ∥}_{\infty }\leq \epsilon }L\left(f_{\theta }\left(P(X+\delta )\right),y\right), \end{array}$$

where gradients are propagated through both the purifier and classifier. This evaluation assesses whether the observed robustness persists when the attacker optimizes through the complete defended pipeline.

In our experiments, adaptive FGSM and adaptive PGD attacks are generated by computing gradients through the entire purification-classification pipeline, thereby representing a stronger adaptive white-box adversary than standard attacks that target the classifier alone.

## 4. Proposed Methodology: ADS-Guard Framework

This section presents ADS-Guard, an *Adversarial Defense and Sanitization* framework designed to defend deep occupancy detection models against adversarial attacks targeting multivariate time-series sensor data [27,28]. ADS-Guard operates as an input-level purification module placed in front of any downstream classifier, making it reusable across multiple classifier architectures and easily deployable across heterogeneous smart building systems.

The core idea of ADS-Guard is to learn a sequence-to-sequence transformation that removes adversarial perturbations while preserving the semantic and temporal structure of the original signal. To achieve this, we employ a recurrent autoencoder trained not only to reconstruct clean inputs but also to enforce *latent consistency* between clean and adversarial representations [17,33].

### 4.1. Overall Pipeline

Given an input sensor sequence $X\in R^{T\times F}$, ADS-Guard applies a purification function P(·) before the sequence is passed to the downstream classifier, $f_{\theta }\left(\cdot \right)$. At inference time, the decision process becomes

(14) $$\begin{array}{c} \hat{y}=f_{\theta }\left(P\left(X\right)\right), \end{array}$$

where P is implemented by a trained sequence-to-sequence autoencoder [17]. For clean inputs, P(X)≈X, while for adversarial inputs $X^{adv}$, the purifier aims to remove adversarial artifacts and produce a purified sequence $\tilde{X}$ that is close to the original clean signal in both signal space and feature space [31,32].

The downstream classifier $f_{\theta }$ can be any temporal model. In this work, we evaluate five representative architectures, LSTM, GRU, 1D-CNN, MLP, and Transformer, without modifying their internal structure or training procedure [22,23,24,25].

### 4.2. Sequence-to-Sequence Autoencoder Architecture

ADS-Guard is built upon a recurrent sequence-to-sequence autoencoder composed of an encoder, $E_{\phi }\left(\cdot \right)$, and a decoder, $D_{\psi }\left(\cdot \right)$ [17,33]. Given an input sequence, X, the encoder maps it to a compact latent representation

(15) $$\begin{array}{c} z=E_{\phi }\left(X\right), \end{array}$$

where $z\in R^{d}$ and d≪T×F. The decoder then reconstructs the sequence as

(16) $$\begin{array}{c} \hat{X}=D_{\psi }\left(z\right)=D_{\psi }\left(E_{\phi }\left(X\right)\right). \end{array}$$

In our implementation, both the encoder and decoder are realized using stacked LSTM layers, while the specific architectural hyperparameters are described in Section 5 [23,24,41]. The encoder compresses the temporal sequence into a fixed-dimensional latent vector, while the decoder expands this representation back into a sequence of length *T* using a repeat-and-decode strategy. A time-distributed linear layer produces the final reconstructed sensor features at each time step.

### 4.3. Reconstruction Objective

The primary training objective of the autoencoder is to minimize the reconstruction error between the input sequence and its reconstruction. This is formulated using the mean squared error (MSE) loss:

(17) $$\begin{array}{c} L_{rec}=\frac{1}{TF}\sum_{t=1}^{T}\sum_{f=1}^{F}{\left(X_{t,f}-{\hat{X}}_{t,f}\right)}^{2}. \end{array}$$

Minimizing $L_{rec}$ ensures that the autoencoder preserves the essential temporal and cross-sensor structure of clean input sequences [33].

### 4.4. Latent Consistency Regularization

While a standard autoencoder can denoise mild stochastic noise, it is insufficient to counteract adversarial perturbations explicitly optimized to fool a classifier [31,33]. To address this limitation, ADS-Guard introduces a *Latent Consistency Regularization* objective.

Given a clean input sequence X and its adversarial counterpart $X^{adv}$, we enforce their latent representations to be close in the feature space:

(18) $$\begin{array}{c} z=E_{\phi }\left(X\right),\;z^{adv}=E_{\phi }\left(X^{adv}\right). \end{array}$$

The latent consistency loss is defined as

(19) $$\begin{array}{c} L_{lat}={\left||z-z^{adv}\right||}_{2}^{2}. \end{array}$$

This regularization term encourages the encoder to learn representations that are less sensitive to adversarial perturbations by promoting proximity between clean and adversarial latent embeddings. As a result, the purifier learns feature representations that better preserve the semantic content of the original signal under attack [17,30].

### 4.5. Adversarial Training of ADS-Guard

To train ADS-Guard, we expose the purifier to adversarially perturbed sequences generated using a surrogate classifier (implemented as an LSTM sequence classifier) [26,29]. The purpose of this procedure is not to evaluate robustness but to explicitly teach the autoencoder to remove adversarial perturbations and enforce invariance in the latent space.

For each mini-batch of clean sequences X with labels *y*, we construct adversarial samples $X^{adv}$ by applying a single-step FGSM attack that maximizes the surrogate classifier’s loss under an $\ell_{\infty }$ constraint:

(20) $$\begin{array}{c} X^{adv}=X+\epsilon_{train}\cdot \mathrm{sign}\left(\nabla_{X}L\left(f_{\theta_{s}}\left(X\right),y\right)\right), \end{array}$$

where $f_{\theta_{s}}$ denotes the fixed surrogate classifier, and $\epsilon_{train}$ controls the perturbation magnitude used during defense training [26].

The ADS-Guard autoencoder is then optimized using a joint objective:

(21) $$\begin{array}{c} L_{total}=L_{rec}+\lambda L_{lat}, \end{array}$$

where Lrec is the reconstruction loss between X and its reconstruction, and $L_{lat}$ enforces latent consistency between X and $X^{adv}$. The weighting coefficient λ controls the trade-off between reconstruction fidelity and adversarial invariance [30].To quantify the contribution of latent consistency regularization, we perform an ablation study across multiple values of λ (Section 6).

Following the ablation study described in Section 6.8, λ=0.01 was selected for all subsequent experiments, which provided a strong balance between reconstruction fidelity and adversarial robustness. Reconstruction-only autoencoders (λ=0) are evaluated separately in the ablation study to quantify the contribution of latent consistency regularization. Importantly, the adversarial examples used in this stage serve only to train the purifier; the evaluation of robustness against FGSM and PGD attacks on the target classifiers is described separately in Section 5.

### 4.6. Inference-Time Purification

At inference time, ADS-Guard operates as a lightweight pre-processing module. Given an input sequence, X, which may be clean or adversarially perturbed, the purified output is computed as

(22) $$\begin{array}{c} \tilde{X}=D_{\psi }\left(E_{\phi }\left(X\right)\right). \end{array}$$

This sanitized sequence is then passed to the downstream classifier:

(23) $$\begin{array}{c} \hat{y}=f_{\theta }\left(\tilde{X}\right). \end{array}$$

Because ADS-Guard is trained to align the latent representations of clean and adversarial inputs, the classifier receives inputs that are generally closer to the clean data manifold. This improves robustness against adversarial perturbations without requiring modifications to the downstream classifier [31,32].

### 4.7. Adaptive Evaluation Setting

Although ADS-Guard is trained on adversarial examples generated by a surrogate LSTM classifier, its robustness evaluation is not limited to attacks against the surrogate model. During testing, we evaluate ADS-Guard under adaptive white-box attacks that explicitly optimize through the combined purification-classification pipeline. In this setting, gradients are propagated through both the purifier and downstream classifier, enabling the attacker to target the deployed defense mechanism directly. This evaluation provides a more robust assessment of robustness by allowing the attacker to optimize through the complete defended pipeline.

### 4.8. Classifier—Independent Deployment

A key advantage of ADS-Guard is its architecture-agnostic design. Since the purification module operates purely on the input space, it can be seamlessly integrated with any temporal classifier, including LSTM, GRU, CNN-based, MLP-based, and Transformer-based models [22,23,24,25,42]. This makes ADS-Guard particularly suitable for real-world smart building deployments, where heterogeneous models may coexist across edge and cloud platforms. Unlike model-hardening defenses such as adversarial training or ensemble diversification [43], which require retraining each target model separately, ADS-Guard can be integrated as an independent preprocessing stage and reused across multiple classifier architectures. For each dataset, a single ADS-Guard purifier is trained once and subsequently reused with all downstream classifier architectures evaluated in this work.

In the next section, we describe the datasets, preprocessing steps, training protocols, and evaluation metrics used to evaluate ADS-Guard across multiple architectures, datasets, and adversarial threat scenarios.

## 5. Experimental Setup

This section describes the datasets, preprocessing pipeline, model configurations, adversarial attack settings, and evaluation metrics used to assess ADS-Guard. The experimental design evaluates robustness across three dimensions: (i) dataset domain, (ii) label structure (binary versus multi-class), and (iii) classifier architecture. Unless otherwise specified, all experiments use fixed random seeds to ensure reproducibility [23,25].

### 5.1. Datasets and Task Definitions

We evaluate ADS-Guard using three publicly available datasets that represent different occupancy-detection scenarios and learning challenges.

#### 5.1.1. Dataset 1: UCI Occupancy Detection (Binary)

The first dataset is the widely used UCI Occupancy Detection dataset [18], consisting of environmental sensor measurements annotated with binary occupancy labels (occupied or empty). We use the standard three-file split provided with the dataset: datatraining, datatest, and datatest2. This predefined split enables direct comparison with previous occupancy-detection studies while avoiding data leakage between training and evaluation sets.

#### 5.1.2. Dataset 2: Room Occupancy (Binary, Imbalanced)

The second dataset (room_occupancy.csv) [20] represents a binary occupancy-detection problem derived from occupancy-count measurements. We convert Room_Occupancy_Count into a binary label:

(24) $$\begin{array}{c} y=I[\mathrm{Room}\_\mathrm{Occupancy}\_\mathrm{Count}>0], \end{array}$$

where I[·] denotes the indicator function. The resulting dataset exhibits substantial class imbalance, with occupied samples occurring much less frequently than empty-room observations. To mitigate this imbalance, class weights are computed from the training partition and incorporated during classifier training.

#### 5.1.3. Dataset 3: Building59 (Multi-Class: Low/Medium/High)

The third dataset (building59_data_extended.csv) models building occupancy [19] as a three-class classification problem with occupancy levels {Low, Medium, High}. These categories are mapped to integer labels:

Low→0,Medium→1,High→2.

Unlike the previous datasets, this dataset contains measurements collected from multiple buildings. To preserve temporal consistency, sequences are generated independently for each building rather than across the entire dataset, ensuring that no sequence spans observations from different buildings.

### 5.2. Preprocessing and Sequence Construction

#### 5.2.1. Feature Selection and Normalization

Let $x_{t}\in R^{F}$ denote the feature vector at time *t*. For each dataset, non-feature fields such as timestamps and identifiers are removed before preprocessing. For the UCI Occupancy dataset, the official test files were retained unchanged. In contrast, the official training file was chronologically divided into internal training and validation partitions before normalization and sequence generation. For the Room Occupancy and Building59 datasets, preprocessing is performed directly on the raw time series.

To prevent temporal leakage between training and evaluation, the raw observations are first partitioned into mutually exclusive contiguous data blocks. These blocks are then assigned to the training, validation, and testing partitions before any sliding-window sequences are generated. Standardization statistics are computed using only the training partition and subsequently applied to the validation and test partitions.

For each feature, *f*, z-score normalization is performed as

(25) $$\begin{array}{c} {\tilde{x}}_{t}^{\left(f\right)}=\frac{x_{t}^{\left(f\right)}-\mu_{f}}{\sigma_{f}}, \end{array}$$

where $\mu_{f}$ and $\sigma_{f}$ denote the mean and standard deviation estimated exclusively from the training partition. This protocol prevents information leakage while improving optimization stability across classifier architectures. For the Building59 dataset, the categorical variable Building_Type is one-hot encoded before normalization.

#### 5.2.2. Sliding Window Sequence Generation

Occupancy prediction is formulated as a multivariate sequence-classification problem using fixed-length sliding windows [22,24]. After the temporal partitions have been established, sequences are generated independently within each partition. A window length of T=20 and a step size of s=1 are used throughout this work.

Given a normalized time series, $\left\{{\tilde{x}}_{1},\ldots ,{\tilde{x}}_{N}\right\}$, the *i*-th sequence is constructed as

(26) $$\begin{array}{c} X_{i}=\left[{\tilde{x}}_{i},{\tilde{x}}_{i+1},\ldots ,{\tilde{x}}_{i+T-1}\right]\in R^{T\times F}. \end{array}$$

The corresponding sequence label is assigned using the occupancy label at the final time step,

(27) $$\begin{array}{c} y_{i}=y_{i+T-1}. \end{array}$$

Generating sequences only after the temporal partitioning ensures that overlapping windows never appear across different data splits, thereby eliminating the sequence-level data leakage that can arise when sliding windows are generated before dataset partitioning.

#### 5.2.3. Per-Building Sequence Construction

The Building59 dataset contains measurements collected from multiple buildings with independent temporal dynamics. To preserve temporal consistency, the data are first grouped by Building_ID, and contiguous temporal blocks are created independently within each building. Sliding-window sequences are then generated separately inside each partition for every building,

(28) $$\begin{array}{c} {\left\{X_{i}^{\left(b\right)},y_{i}^{\left(b\right)}\right\}}_{i=1}^{n_{b}}, \end{array}$$

where *b* denotes an individual building. The resulting sequences from all buildings are concatenated after partitioning to form the final training, validation, and testing sets.

This procedure guarantees that each sequence originates from a single building and that no overlapping temporal observations are shared across different evaluation partitions.

### 5.3. Data Splits and Class Imbalance Handling

#### 5.3.1. Predefined Benchmark Splits and Internal Validation (UCI)

For the UCI Occupancy dataset, the official files datatraining, datatest, and datatest2 were used. The datatest and datatest2 files were retained as the two independent test partitions, denoted Test1 and Test2, respectively.

The official datatraining file was further divided chronologically into internal training and validation partitions. The first 90% of the observations were assigned to the training portion and the remaining 10% to validation. Because the samples were subsequently converted into overlapping sliding-window sequences of length T=20, a purge interval of T−1=19 observations was removed on both sides of the training–validation boundary. This ensured that no raw observation contributed to sequences in both partitions.

The resulting partitions can be expressed as

$$D_{\mathrm{train}}=\mathrm{datatraining}\left[1:\;k-\left(T-1\right)\right],$$

$$D_{\mathrm{val}}=\mathrm{datatraining}\left[k+\left(T-1\right):\;N\right],$$

where *N* is the number of observations in datatraining and k=⌊0.9N⌋ is the chronological split point.

The feature scaler was fitted exclusively on the raw observations in $D_{\mathrm{train}}$ and was then applied without refitting to $D_{\mathrm{val}}$, Test1, and Test2. Sliding-window sequences were generated separately within each partition after scaling. This procedure prevents both normalization leakage and overlap between training and validation sequences while preserving the official UCI test sets for final evaluation.

#### 5.3.2. Temporal Block Partitioning (Room Occupancy and Building59)

For the Room Occupancy and Building59 datasets, the raw time series is first divided into mutually exclusive contiguous temporal blocks. These blocks are assigned to the data partitions before sliding-window sequences are generated. Consequently, all overlapping windows originating from a given block remain within the same partition, and no raw observation is shared across training, validation, Test1, or Test2.

Blocks are allocated using a 60%/10%/10%/20% split for training, validation, Test1, and Test2, respectively. The validation partition is used exclusively for early stopping and model selection, whereas Test1 and Test2 are held out from training and are used only for final clean and adversarial evaluation. Block allocation is stratified according to the block-level label distribution to preserve class representation across partitions as closely as permitted by the available number of blocks.

For the Building59 dataset, block construction and allocation are performed independently within each building before sequences from the assigned blocks are combined. This prevents sequences from crossing building boundaries while evaluating temporally distinct segments from the available buildings. The resulting protocol is a within-building temporal evaluation and should not be interpreted as a held-out-building generalization experiment.

#### 5.3.3. Class Weights

To mitigate class imbalance, class weights are computed using only the training partition and incorporated into classifier training. For *C* classes, let $n_{c}$ denote the number of training samples belonging to class *c*, and let $N=\sum_{c=1}^{C}n_{c}$. The balanced class weight is computed as

(29) $$\begin{array}{c} w_{c}=\frac{N}{C\;n_{c}}. \end{array}$$

During training, each sample contributes a weighted loss of $w_{y_{i}}L\left(\cdot \right)$, reducing the bias toward majority classes and improving learning stability under imbalanced class distributions. For the binary occupancy datasets, C=2, while for the Building59 dataset, C=3.

### 5.4. Models and Training Protocol

#### 5.4.1. Base Classifiers

We evaluate five deep sequence classifiers commonly used in time series prediction:

- **LSTM:** stacked LSTM layers with dropout, followed by a dense output layer.
- **GRU:** stacked GRU layers with dropout, followed by a dense output layer.
- **1D-CNN:** temporal convolution layers, pooling, and then global average pooling.
- **MLP:** flattened sequence input followed by fully connected layers.
- **Transformer:** feature projection, positional embedding, multi-head self-attention, feed-forward layers, and then pooling.

##### Binary Tasks

For binary occupancy detection (UCI, Room Occupancy), classifiers output a scalar probability:

(30) $$\begin{array}{c} p_{\theta }\left(y=1|X\right)=\sigma \left(g_{\theta }\left(X\right)\right), \end{array}$$

where σ(·) is the sigmoid function. We train with binary cross-entropy:

(31) $$\begin{array}{c} L_{bin}=-\left[y\log(p)+(1-y)\log(1-p)\right]. \end{array}$$

##### Multi-Class Task

For the Building59 dataset, classifiers output a probability vector:

(32) $$\begin{array}{c} p_{\theta }\left(X\right)\in {\left[0,1\right]}^{C},\;\sum_{c=1}^{C}p_{c}=1, \end{array}$$

using a softmax output. We train with sparse categorical cross-entropy:

(33) $$\begin{array}{c} L_{mc}=-\log\left(p_{y}\right). \end{array}$$

#### 5.4.2. Optimization and Regularization

All classifiers are trained using the Adam optimizer with a learning rate of $10^{-3}$. Dropout (0.3) is employed to reduce overfitting, and early stopping monitors the validation loss with a patience of five epochs while restoring the best-performing model weights.

Training is performed using mini-batch optimization. The downstream classifiers are trained with a batch size of 64 for the UCI and Room Occupancy datasets and 128 for the Building59 dataset. Validation data are supplied explicitly from the predefined validation partition during training. Early stopping monitored validation loss without using the held-out test partitions. This internal validation subset is derived exclusively from the training partition and is never mixed with the held-out Test1 or Test2 evaluation sets. Random seeds for NumPy and TensorFlow are fixed to ensure reproducibility. For each dataset, a single ADS-Guard purifier is trained using the corresponding training partition. The trained purifier is then reused with each downstream classifier architecture without additional retraining.

#### 5.4.3. ADS-Guard Purifier Configuration

ADS-Guard is implemented as a sequence-to-sequence recurrent autoencoder with:

- Encoder: stacked LSTM layers compressing $X\in R^{T\times F}$ to a latent vector $z\in R^{d}$.
- Latent dimension: d=32.
- Decoder: RepeatVector followed by stacked LSTM layers and a time-distributed output head.

##### Standard Purification Baseline

A baseline purifier is trained using reconstruction loss only:

(34) $$\begin{array}{c} L_{total}=L_{rec}. \end{array}$$

##### ADS-Guard with Latent Consistency

In the full ADS-Guard setting, the purifier is trained with both reconstruction and latent consistency:

(35) $$\begin{array}{c} L_{total}=L_{rec}+\lambda L_{lat}. \end{array}$$

Here, λ controls the trade-off between reconstruction fidelity and latent-space consistency. Based on the ablation study presented in Section 6, all experiments use λ=0.01, which provided the strongest overall robustness under both standard and adaptive attacks.

### 5.5. Adversarial Attack Configuration

We evaluate robustness under two white-box, untargeted adversarial attacks that constrain perturbations under an $\ell_{\infty }$ budget ϵ:

(36) $$\begin{array}{c} {∥\delta ∥}_{\infty }\leq \epsilon . \end{array}$$

We sweep ϵ∈{0.1,0.2,0.3,0.5} to characterize robustness under increasing adversarial strength. These perturbation magnitudes are applied to standardized input features following the preprocessing procedure described in Section 5.2.1.

#### 5.5.1. FGSM

FGSM constructs adversarial inputs in a single step:

(37) $$\begin{array}{c} X^{adv}=X+\epsilon \cdot \mathrm{sign}\left(\nabla_{X}L\left(f_{\theta }\left(X\right),y\right)\right). \end{array}$$

For binary tasks, L is binary cross-entropy. For multi-class tasks, L is sparse categorical cross-entropy.

#### 5.5.2. PGD

PGD iteratively refines adversarial inputs with projection back into the $\ell_{\infty }$ ball:

(38) $$\begin{array}{c} X_{k+1}^{adv}=\Pi_{B_{\epsilon }\left(X\right)}\left(X_{k}^{adv}+\alpha \cdot \mathrm{sign}\left(\nabla_{X}L\left(f_{\theta }\left(X_{k}^{adv}\right),y\right)\right)\right). \end{array}$$

For standard robustness evaluation, PGD attacks are generated using five iterative updates. To provide a stronger robustness assessment, additional experiments employ 20-step PGD attacks as well as adaptive PGD attacks that explicitly optimize through the ADS-Guard purification module. Unless otherwise specified, the step size is set as α=1.5ϵ/K with K=5 iterations, matching our implementation and ensuring stable projected updates within the $\ell_{\infty }$ budget.

### 5.6. Adaptive White-Box

To evaluate robustness against a defense-aware adversary, we additionally consider adaptive white-box attacks. Unlike standard FGSM and PGD attacks that optimize perturbations against the classifier alone, adaptive attacks optimize perturbations against the complete purification-classification pipeline.

Let P(·) denote ADS-Guard, and let $f_{\theta }\left(\cdot \right)$ denote the downstream classifier. Adaptive attacks maximize

(39) $$\begin{array}{c} L\left(f_{\theta }\left(P\left(X+\delta \right)\right),y\right), \end{array}$$

subject to the same $\ell_{\infty }$ perturbation budget used throughout this study. Gradients are propagated through both the purifier and the downstream classifier, providing a stronger evaluation of defense robustness under a defense-aware white-box adversary.

### 5.7. Adversarial Training Baselines

To benchmark ADS-Guard against a widely adopted adversarial defense strategy, we additionally train classifiers using FGSM-based adversarial training (FGSM-AT) and PGD-based adversarial training (PGD-AT). During training, adversarial examples are generated online and incorporated into the optimization process.

FGSM-AT uses single-step FGSM perturbations during training, whereas PGD-AT employs iterative PGD perturbations. The adversarially trained models are evaluated under the same attack settings used for ADS-Guard, enabling a direct comparison between retraining-based defenses and the proposed retraining-free purification framework.

### 5.8. Evaluation Metrics

We report performance on clean, attacked, and purified inputs using Accuracy, F1-score, Precision, Recall, and AUROC. Metrics are computed separately on test1 and test2.

#### 5.8.1. Binary Metrics

Given predicted labels ${\hat{y}}_{i}\in \left\{0,1\right\}$ and ground truth labels $y_{i}\in \left\{0,1\right\}$, we compute:

(40) $$\begin{array}{c} \mathrm{Accuracy}=\frac{1}{N}\sum_{i=1}^{N}I\left[{\hat{y}}_{i}=y_{i}\right], \end{array}$$

(41) $$\begin{array}{c} \mathrm{Precision}=\frac{TP}{TP+FP} \end{array}$$

(42) $$\begin{array}{c} \mathrm{Recall}=\frac{TP}{TP+FN}, \end{array}$$

(43) $$\begin{array}{c} F1=\frac{2\cdot \mathrm{Precision}\cdot \mathrm{Recall}}{\mathrm{Precision}+\mathrm{Recall}}. \end{array}$$

AUROC is computed from predicted probabilities $p_{i}=p_{\theta }\left(y=1|X_{i}\right)$ and reflects the ranking quality across decision thresholds.

#### 5.8.2. Multi-Class Metrics

For the multi-class task (C=3), we compute weighted Precision, Recall, and F1 across classes:

(44) $$\begin{array}{c} {F1}_{weighted}=\sum_{c=1}^{C}\frac{n_{c}}{N}{F1}_{c}, \end{array}$$

and similarly for Precision and Recall, where $n_{c}$ is the number of test samples in class *c*.

For AUROC, we adopt a one-vs-rest (OvR) strategy using the predicted probability vector $p_{i}$:

(45) $$\begin{array}{c} {\mathrm{AUROC}}_{OvR}=\sum_{c=1}^{C}\frac{n_{c}}{N}\mathrm{AUROC}\left(I\left[y=c\right],\;p^{\left(c\right)}\right), \end{array}$$

where $p^{\left(c\right)}$ is the predicted probability of class *c*. This provides a robust threshold-independent evaluation for multi-class classification.

### 5.9. Evaluation Protocol

For each dataset and classifier architecture, we report:

- Clean performance on unperturbed inputs;
- Performance under FGSM and PGD attacks without defense;
- Performance after ADS-Guard purification;
- Robustness under adaptive white-box attacks;
- Performance of FGSM-based and PGD-based adversarial-training baselines.

This evaluation protocol enables direct comparison between retraining-free purification and retraining-based defense strategies across diverse datasets, architectures, and attack settings.

## 6. Results

We evaluate ADS-Guard on three datasets under both FGSM and PGD attacks, spanning binary and multi-class occupancy inference and five different classifier architectures (LSTM, GRU, CNN1D, MLP, Transformer). For each setting, we report performance on clean inputs, adversarial inputs without defense, and adversarial inputs after purification. The results are reported on the more challenging test2 split. In the main paper, we focus on a representative strong attack regime (ϵ=0.3 for UCI and Room, ϵ=0.2 for Building59).

### 6.1. Overall Experimental Performance

The proposed ADS-Guard framework was evaluated on three occupancy prediction benchmarks representing progressively different learning scenarios: the UCI Occupancy Detection dataset, the Room Occupancy dataset, and the Building59 dataset. Together, these datasets span binary and multi-class classification tasks while varying considerably in complexity, temporal characteristics, and deployment realism. Performance was assessed using five deep learning architectures (LSTM, GRU, 1D-CNN, MLP, and Transformer) under clean conditions, standard white-box adversarial attacks (FGSM and PGD), adaptive white-box attacks, and adversarial-training baselines.

Across all datasets, the evaluated classifiers achieved strong performance on clean data, although accuracy varied with dataset complexity. As expected, adversarial perturbations consistently reduced classification performance, with larger perturbation budgets producing greater degradation. Applying ADS-Guard before classification improved robustness across the evaluated architectures by mitigating the effect of adversarial perturbations while preserving competitive clean-data performance.

The Building59 benchmark proved substantially more challenging than the binary occupancy datasets because of its chronological evaluation protocol, multi-sensor data integration, and three-class occupancy prediction task. Consequently, absolute classification performance on this dataset was lower for all evaluated models. Nevertheless, ADS-Guard continued to improve robustness under both standard and adaptive adversarial attacks without requiring retraining of the downstream classifier.

The following subsections provide a detailed analysis of performance on each dataset, followed by comparisons under adaptive attacks, adversarial-training baselines, additional evaluation metrics, and the influence of the latent consistency coefficient.

### 6.2. Dataset 1: UCI Occupancy Detection (Binary, ϵ=0.3)

Based on the ablation analysis, the latent consistency coefficient was set to λ=0.01 for the UCI dataset experiments. Although robustness was evaluated using ϵ∈{0.1,0.2,0.3,0.5}, Table 3 reports Test2 accuracy at ϵ=0.3 as a representative perturbation setting. The complete perturbation sweep is reported separately.

As shown in Table 3, both FGSM and PGD reduced the accuracy of all evaluated classifiers. Applying ADS-Guard improved accuracy under both attacks for every architecture. The largest improvements were observed for the MLP, increasing from 0.7290 to 0.9180 under FGSM and from 0.6966 to 0.9229 under PGD. ADS-Guard also improved the LSTM from 0.7090 to 0.9050 under FGSM and from 0.7119 to 0.8706 under PGD. Although the magnitude of improvement varied across architectures, ADS-Guard consistently improved classification accuracy relative to the corresponding undefended FGSM and PGD results.

### 6.3. Dataset 2: Room Occupancy (Binary, ϵ=0.3)

Based on the ablation study, we selected λ=0.01 for the Room Occupancy Dataset because it provided the best balance between preserving clean performance and improving adversarial robustness across multiple architectures. We further selected an attack strength of ϵ=0.3, as smaller perturbations produced only minor degradation, while larger perturbations (ϵ=0.5) resulted in unrealistically severe attacks. Thus, ϵ=0.3 provides a more representative evaluation of defense performance under moderate adversarial perturbations.

Table 4 reports the classification accuracy under FGSM and PGD attacks before and after applying ADS-Guard. The proposed defense substantially restored performance for most architectures under both attack types. In particular, MLP, GRU, and Transformer consistently exhibited large improvements after purification, while CNN1D exhibited comparatively smaller improvements. The LSTM model exhibited only limited improvement because its baseline adversarial accuracy remained high even without defense, leaving relatively little room for recovery.

### 6.4. Dataset 3: Building59 (Multi-Class, ϵ=0.2)

Based on the ablation study, λ=0.01 was selected, as it provides the best balance between clean performance and adversarial robustness. For the Building59 dataset, we report results using an attack strength of ϵ=0.2, representing a moderate perturbation where the degradation caused by the attack is substantial while still allowing meaningful recovery through ADS-Guard.

Table 5 summarizes the performance of ADS-Guard across five temporal architectures. Without defense, both FGSM and PGD attacks substantially reduce classification performance across all models. After purification, ADS-Guard consistently improves both Accuracy and F1-score. For example, the LSTM accuracy increases from 37.28% to 50.38% under FGSM and from 37.07% to 51.66% under PGD. The MLP achieves the largest recovery, improving from 27.78% to 55.36% under FGSM and from 25.20% to 55.75% under PGD, while its F1-score increases from 0.238 to 0.543 and from 0.210 to 0.547, respectively. Similar improvements are observed for GRU, CNN1D, and Transformer models, demonstrating that ADS-Guard consistently restores predictive performance across diverse temporal architectures under both single-step and iterative adversarial attacks.

### 6.5. Adaptive White-Box Evaluation

To further assess the robustness of ADS-Guard, we evaluated the defense under adaptive white-box attacks in which adversarial examples were generated by propagating gradients through the purification module. Unlike the standard FGSM and PGD attacks, the adaptive attacker explicitly accounts for the defense during optimization, representing a substantially stronger threat model. Based on the ablation study, λ=0.01 was used for all datasets, with ϵ=0.3 for the UCI and Room Occupancy Datasets and ϵ=0.2 for the Building59 dataset.

Table 6 summarizes the classification accuracy under both standard and adaptive attacks. As expected, Adaptive attacks generally altered the defended performance relative to standard attacks, although the magnitude of the change varied across architectures and datasets. In most cases, adaptive attacks reduced classification accuracy. At the same time, a small number of models exhibited comparable or slightly improved performance, reflecting the optimization dynamics of the adaptive attack rather than a systematic improvement in robustness.

Small increases observed for a few architectures are attributable to the non-convex optimization landscape of the defended pipeline and should not be interpreted as adaptive attacks improving robustness. Overall, adaptive attacks represent a stronger evaluation setting and generally reduce defended performance.

### 6.6. Comparison with Adversarial Training

To further assess the proposed defense, ADS-Guard was compared with adversarial training (AT), one of the most widely adopted strategies for improving robustness against adversarial attacks. Both FGSM and PGD-based adversarial training were implemented using the same classifier architectures and training protocol employed throughout this study. During evaluation, the adversarially trained models were tested using the same attack settings selected for each dataset (ϵ=0.3 for the UCI and Room Occupancy Datasets and ϵ=0.2 for the Building59 dataset). Since PGD-based adversarial training consistently outperformed FGSM-based adversarial training across the evaluated architectures, only the stronger PGD-AT baseline is reported in Table 7.

On the UCI dataset, PGD-AT generally achieved higher attack accuracy for the recurrent architectures, whereas ADS-Guard provided comparable performance for the MLP and Transformer models. For the Room Occupancy Dataset, both defenses substantially improved robustness, with PGD-AT performing better for the GRU and CNN1D models. At the same time, ADS-Guard achieved comparable or slightly higher robustness for the LSTM and MLP architectures. On the more challenging Building59 dataset, neither defense consistently dominated across all architectures. ADS-Guard achieved higher attack accuracy for the LSTM and MLP models. In contrast, PGD-AT provided better robustness for the GRU and Transformer models, and both methods produced comparable performance for CNN1D.

These observations indicate that ADS-Guard achieves robustness comparable to adversarial training while requiring only a single purification model that can be integrated with multiple downstream classifiers. In contrast, adversarial training requires each classifier to be retrained individually using adversarial examples. This classifier-independent design makes ADS-Guard easier to deploy while maintaining competitive robustness across diverse network architectures and occupancy datasets.

### 6.7. Summary of Findings

The experimental evaluation across three occupancy detection datasets demonstrates that ADS-Guard consistently improves robustness against adversarial perturbations while maintaining high predictive performance on clean data. Across the UCI, Room, and Building59 datasets, the proposed purification framework effectively mitigated the impact of both FGSM and PGD attacks for multiple sequence modeling architectures, including LSTM, GRU, CNN1D, MLP, and Transformer models.

The adaptive white-box evaluation further showed that, although attack effectiveness increased when the adversary optimized through the complete defended pipeline, ADS-Guard continued to preserve meaningful predictive performance, indicating that the learned latent representations remain effective even under stronger adaptive attacks. Comparison with adversarial training demonstrated that ADS-Guard achieves competitive robustness while avoiding classifier-specific retraining, allowing a single purification model to be integrated with multiple downstream classifiers.

The ablation study highlighted the importance of the latent consistency objective, with λ=0.01 providing the best balance between reconstruction fidelity and adversarial robustness across all evaluated datasets. Additional evaluation using complementary metrics, including F1-score, precision, recall, and AUROC, further confirmed that the observed robustness improvements were consistent beyond overall classification accuracy.

### 6.8. Additional Robustness Metrics

Although classification accuracy provides an intuitive measure of defense effectiveness, it does not fully capture performance under adversarial perturbations, particularly when the class distribution becomes skewed after an attack. Therefore, we further evaluated ADS-Guard using F1-score, precision, recall, and AUROC under the selected attack configurations for each dataset (ϵ=0.3 for the UCI and Room Occupancy Datasets and ϵ=0.2 for the Building59 dataset). These complementary metrics provide a more comprehensive assessment of classification quality by jointly considering false positives, false negatives, and ranking performance.

Table 8 summarizes the results for the representative LSTM model. Similar qualitative trends were observed across the remaining architectures (results omitted for brevity). Across all three datasets, adversarial attacks consistently reduced F1-score, precision, recall, and AUROC, indicating degradation in both classification accuracy and class discrimination. After applying ADS-Guard, all evaluation metrics improved substantially relative to the attacked models. The improvements in F1-score demonstrate that the proposed purification framework preserves balanced classification performance. At the same time, the consistently higher AUROC values indicate that ADS-Guard maintains strong discriminative capability even under adversarial perturbations. These observations are consistent with the accuracy-based analysis presented in the previous sections and confirm that the proposed defense improves robustness without benefiting only a single evaluation metric.

### 6.9. Latent Consistency Ablation

The latent consistency loss encourages similar latent representations for clean and adversarial inputs during autoencoder training. To evaluate its contribution, the weighting parameter λ was varied over {0,0.01,0.05,0.1,0.5} while keeping all other training settings unchanged. For each value of λ, the complete experimental pipeline was repeated on the UCI, Room, and Building59 datasets.

The evaluation considered clean-data performance together with robustness under FGSM and PGD attacks after purification. In addition to classification accuracy, other metrics including F1-score, precision, recall, and AUROC were examined to provide a comprehensive assessment of each configuration. The selected λ was, therefore, determined based on its overall performance across all evaluation metrics and datasets rather than a single performance measure.

Across the three occupancy datasets, λ=0.01 consistently provided the best overall balance between clean-data performance and adversarial robustness while maintaining stable improvements across the complementary evaluation metrics. Accordingly, λ=0.01 was adopted for all subsequent experiments, including the adaptive white-box evaluation, comparison with adversarial training, and additional robustness analysis.

## 7. Conclusions

This paper presented ADS-Guard, an input-level adversarial purification framework for robust occupancy inference from multivariate environmental sensor data in smart building environments. ADS-Guard employs a sequence-to-sequence autoencoder with latent consistency regularization to remove adversarial perturbations before the data reaches the downstream classifier. Because the defense operates entirely in the input space, it can be integrated with existing occupancy-detection systems without modifying or retraining deployed models.

ADS-Guard was evaluated on three occupancy-related datasets representing both binary and multi-class classification tasks using five representative deep learning architectures: LSTM, GRU, CNN1D, MLP, and Transformer. The experimental results demonstrated that adversarial attacks substantially degrade occupancy inference performance under both FGSM and PGD attacks. Across all datasets, ADS-Guard consistently improved robustness while maintaining strong performance on clean data, with particularly effective recovery on the binary occupancy datasets and meaningful robustness gains on the more challenging multi-class Building59 dataset.

The proposed framework was further evaluated under adaptive white-box attacks in which the adversary optimized through the complete defended pipeline. Although these stronger attacks reduced performance compared with the standard attack setting, ADS-Guard continued to preserve meaningful predictive performance, demonstrating that the learned latent representations provide robustness beyond the attack scenarios used during training. A comparison with adversarial training further showed that ADS-Guard achieves competitive robustness while offering an important practical advantage: a single purification model can be integrated with multiple downstream classifiers without requiring classifier-specific retraining.

A comprehensive ablation study investigated the effect of the latent consistency regularization weight by evaluating the complete experimental pipeline across multiple λ values on all three datasets. Based on clean performance, adversarial robustness, and complementary evaluation metrics, λ=0.01 was selected as the default configuration, providing the best overall balance between reconstruction fidelity and robustness. These findings demonstrate that latent consistency regularization is a key component of the proposed purification framework.

Overall, ADS-Guard provides an effective and flexible defense against adversarial attacks for deep-learning-based occupancy detection. By combining architecture-independent deployment with consistent robustness improvements across multiple datasets, attack settings, and model architectures, the proposed framework offers a practical solution for enhancing the security and reliability of intelligent smart-building systems.

Future work will investigate stronger adaptive and black-box attack strategies, evaluate transferability across unseen buildings and sensing environments, and assess computational overhead and scalability in resource-constrained edge deployments. We also plan to explore the integration of purification-based defenses with uncertainty estimation and adversarial detection techniques further to improve the resilience of intelligent building management systems.

## Figures and Tables

**Table 1:** Part A: Comparative Analysis of Adversarial Security across Time-Series Domains.

Domain	Typical Modality	Primary Attack Goal	Current Defence Status	Relevance to Occupancy
Medical Diagnostics	ECG/EEG Signals	Misdiagnosis (e.g., hiding arrhythmia) [34]	High: Certified robustness and domain-specific smoothing	Shows 1D biological signals are highly vulnerable to PGD.
Smart Grids	Power Consumption	Load Alteration (e.g., causing blackouts) [35]	Moderate: Anomaly detection for "False Data Injection"	Analogous to "Energy Sabotage" in buildings.
Human Activity (HAR)	Accelerometer/Gyro	Evasion (e.g., faking inactivity) [36]	Low: Mostly focuses on random noise, not adversarial	Closest proxy to occupancy; high failure rate under PGD.
Occupancy Detection	Environmental Sensors (Temp/CO_2_)	Energy Sabotage and Intrusion	None/Neglected: Models treated as "Black Boxes"	Our Work (ADS-Guard) fills this specific gap.

**Table 2:** Part B: Algorithms, Attacks, and Defenses in the Literature.

Category	Algorithm	Primary Focus	Key Advantages	Critical Limitation in IoT Context
Detection Models	SVM/Random Forest [21]	Binary Classification	Interpretable, low compute.	Requires manual feature engineering; scales poorly.
	CNN/LSTM/GRU [22]	Temporal features	High accuracy on raw data.	Highly Brittle: Vulnerable to imperceptible noise.
	Transformers [25]	Global Dependencies	State-of-the-Art accuracy.	High memory footprint; susceptible to adversarial perturbations.
Attacks	FGSM [26]	Fast Generation	Computationally Cheap	Weak adversary; easily defended by simple training.
	PGD [29]	Iterative Optimization	Strongest first-order attack; verifies worst-case.	Computationally expensive to generate; breaks most defenses.
Defenses	Adversarial Training [29]	Model Hardening	Strong empirical robustness against gradient-based attacks	Requires retraining, access to training data, and attack-specific optimization
	Standard Autoencoders [31]	Denoising (MSE)	Modular defense.	Masking Effect: Reconstructs subtle adversarial noise.
	ADS-Guard (Ours)	Latent Consistency	Classifier-independent purification; Preserves semantic content.	Requires a separate purification pass (slight latency).

**Table 3:** UCI Occupancy Detection Test2 accuracy with λ=0.01 and ϵ=0.3.

Model	Clean	FGSM	FGSM + ADS-Guard	PGD	PGD + ADS-Guard
LSTM	0.8848	0.7090	**0.9050**	0.7119	**0.8706**
GRU	0.9004	0.6950	0.7904	0.7155	0.7999
1D-CNN	0.9041	0.7757	0.8540	0.7670	0.8634
MLP	0.9363	0.7290	0.9180	0.6966	0.9229
Transformer	0.9489	0.8712	0.9102	0.8778	0.9155

**Table 4:** Classification accuracy (%) on the Room Occupancy Dataset using the selected λ=0.01 and ϵ=0.3.

Model	Clean	FGSM Attack	FGSM + ADS-Guard	PGD Attack	PGD + ADS-Guard
LSTM	98.67	96.91	96.00	96.85	95.51
GRU	98.61	89.02	**95.57**	84.60	**94.97**
CNN1D	97.63	95.45	95.51	95.33	95.21
MLP	99.45	89.45	96.60	86.78	96.73
Transformer	98.97	88.54	94.18	77.44	94.00

**Table 5:** Performance on the Building59 dataset using the selected λ=0.01 and ϵ=0.2.

Model	Metric	Clean	FGSM Attack	FGSM + ADS-Guard	PGD Attack	PGD + ADS-Guard
LSTM	Accuracy	65.02	37.28	50.38	37.07	51.66
	F1-score	0.644	0.331	0.490	0.329	0.504
GRU	Accuracy	64.77	39.03	48.05	39.42	48.77
	F1-score	0.644	0.360	0.460	0.365	0.468
CNN1D	Accuracy	57.77	36.88	45.39	35.62	45.96
	F1-score	0.532	0.299	0.424	0.287	0.432
MLP	Accuracy	62.29	27.78	55.36	25.20	55.75
	F1-score	0.607	0.238	0.543	0.210	0.547
Transformer	Accuracy	68.28	36.20	47.84	34.98	47.88
	F1-score	0.679	0.338	0.461	0.323	0.461

**Table 6:** Adaptive white-box evaluation (Accuracy).

Dataset	Model	FGSM + ADS	Adaptive FGSM	PGD + ADS	Adaptive PGD
UCI	LSTM	90.50	87.21	87.06	82.38
	GRU	79.04	82.46	79.99	80.15
	CNN1D	85.40	85.92	86.34	82.19
	MLP	91.80	88.04	92.29	86.26
	Transformer	91.02	93.25	91.55	90.74
Room	LSTM	96.00	92.24	95.51	89.69
	GRU	95.57	91.02	94.97	88.78
	CNN1D	95.51	90.84	95.21	88.36
	MLP	96.60	91.39	96.73	87.57
	Transformer	94.18	90.24	94.00	88.36
Building59	LSTM	50.38	40.00	51.66	38.53
	GRU	48.05	36.08	48.77	34.12
	CNN1D	45.39	36.24	45.96	35.23
	MLP	55.36	39.11	55.75	37.19
	Transformer	47.84	37.46	47.88	35.31

**Table 7:** Comparison of PGD-based adversarial training (PGD-AT) and ADS-Guard using the selected attack settings.

Dataset	Method	LSTM	GRU	CNN1D	MLP	Transformer
UCI	PGD-AT	97.59	97.54	93.94	87.21	97.26
	ADS-Guard	87.06	79.99	86.34	92.29	91.55
Room	PGD-AT	92.66	97.70	97.21	96.91	94.60
	ADS-Guard	95.51	94.97	95.21	96.73	94.00
Building59	PGD-AT	49.08	56.74	46.32	41.61	54.45
	ADS-Guard	51.66	48.77	45.96	55.75	47.88

**Table 8:** Additional robustness metrics for the representative LSTM model using the selected attack settings on each dataset.

Dataset	Scenario	Accuracy	F1-Score	Precision	Recall	AUROC
UCI	Clean	88.48	73.15	71.19	75.22	94.24
	FGSM Attack	70.90	38.14	34.26	43.00	63.81
	FGSM + ADS-Guard	90.50	72.80	90.30	60.99	96.75
	PGD Attack	71.19	39.04	34.94	44.24	65.51
	PGD + ADS-Guard	87.06	65.91	73.18	59.95	94.78
Room	Clean	98.67	90.98	86.05	96.52	99.80
	FGSM Attack	96.91	80.75	71.33	93.04	95.14
	FGSM + ADS-Guard	96.00	74.62	66.90	84.35	98.01
	PGD Attack	96.85	80.45	70.86	93.04	93.89
	PGD + ADS-Guard	95.51	71.76	63.95	81.74	97.66
Building59	Clean	65.02	64.39	65.76	65.02	83.65
	FGSM Attack	37.28	33.12	31.54	37.28	61.14
	FGSM + ADS-Guard	50.38	48.96	50.05	50.38	71.30
	PGD Attack	37.07	32.93	31.44	37.07	61.30
	PGD + ADS-Guard	51.66	50.40	51.39	51.66	72.04

## Data Availability

Data are contained within the article.
